# Supplementary material for: Methionine Restriction Improves Cognitive Ability by Alleviating Hippocampal Neuronal Apoptosis through H19 in Middle-Aged Insulin-Resistant Mice
Source: Nutrients. 2022 Oct 26;14(21):4503. doi: 10.3390/nu14214503 (PMC9653609; doi:10.3390/nu14214503)
Supplement: Supplementary file 1 [file nutrients-14-04503-s001.zip › nutrients-1955000-supplementary.pdf]

Supplementary Materials

Methionine Restriction Improves Cognitive Ability by Alleviating Hippocampal Neuronal Apoptosis through H19 in Middle-Aged Insulin-Resistant Mice

Chuanxing Feng [1,2,†], Yuge Jiang [2,†], Shiying Li [3], Yueting Ge [4], Yonghui Shi [1,2], Xue Tang [1,2] and Guowei Le [1,2,*]

[1] State Key Laboratory of Food Science and Technology, Jiangnan University, Wuxi 214122, China
[2] Center for Food Nutrition and Functional Food Engineering, School of Food Science and Technology, Jiangnan University, Wuxi 214122, China
[3] Key Laboratory of Neuroregeneration of Jiangsu and Ministry of Education, Co-Innovation Center of Neuroregeneration, Nantong University, Nantong 226019, China
[4] College of Life Science, Xinyang Normal University, Xinyang 464000, China
* Correspondence: lgw@jiangnan.edu.cn; Tel.: +86-510-8591-7789
† These authors contributed equally to this work.

Table S1. The compositions of the experimental diets (g/100g)

Ingredient	CON	HF	HFMR
Corn starch	64.09	44.29	44.29
Maltodextrin	5.00	5.00	5.00
Sucrose	0.10	0.10	0.10
Cellulose	5.00	5.00	5.00
CMC	1.00	1.00	1.00
[1]Soy protein	7.59	7.59	7.59
[2]Amino acid mixture	6.35	6.35	6.35
L-Methionine	0.69	0.69	0.00
L-Glutamic acid	1.37	1.37	2.06
Soybean oil	2.00	2.00	2.00
Pork Lard	2.20	22.00	22.00
AIN-76A-Vitamin mixture	1.00	1.00	1.00
AIN-76A-Mineral mixture	3.50	3.50	3.50
Choline chloride	0.11	0.11	0.11
Total	100.00	100.00	100.00

[1] Amino acid composition of soy protein: 6.19% leucine, 4.11% isoleucine, 5.49% valine, 1.18% methionine, 1.66% cysteine, 4.09% phenylalanine, 2.57% tyrosine, 4.83% lysine, 2.21% threonine, 1.07% tryptophan, 1.99% histidine, 6.11% arginine, 3.30% serine, 3.25% alanine, 5.56% proline, 3.27% glycine, 17.49% glutamic acid 9.44% aspartic acid. 1 g cysteine is equal to 0.64 g methionine.

[2] Amino acid mixture: 10.39% l-arginine, 2.83% l-histidine, 8.03% l-isoleucine,10.08% l-leucine, 1.73% l-lysine, 13.39% l-phenylalanine, 10.24% l-threonine, 1.57% l-tryptophan, 8.98% l-valine, 32.76% l-glycine.

Table S2. Sequences of primers used in quantitative real-time reverse transcription PCR

Gene name	Forward primer (5′–3′)	Reverse primer (5′–3′)
H19	GAACAGAAGCATTCTAGGCTGG	TTCTAAGTGAATTACGGTGGGTG
Bcl-2	TCTTTGAGTTCGGTGGGGTCAT	AGACAGCCAGGAGAAATCAAACAGA
Bax	CCAGGATGCGTCCACCAAGA	GCAAAGTAGAAGAGGGCAACCAC
Caspase-3	CTGGAGAAATTCAAAGGACGGG	TGAGCATGGACACAATACACGG
BDNF	AATGGTGTCGTAAAGTTCCAC	GCAACCGAAGTATGAAATAACC
TRκB	AGAACGAGTATGGGAAGGA	TTGGGTTTGTCTCGTAGTC
CAMK2A	AAACACTCAACAAAATCAAACGAC	GCCACAGAGAGACCAAAAGCA
CREB	GAAGAGGAGACTTCAGCCC	TAATGGCAATGTACTGCCCA
Synpo	CCTGCCCGTAACTTCCGTG	GAGCGGCGGTAGGGAAAAG
IRS-1	GCAGCAGTAGCAGCATCAG	TACCGCCACCACTCTCAAC
GLUT1	CAGTTCGGCTATAACACTGGTG	GCCCCCGACAGAGAAGATG
HK2	TGCTGCCGACCTTTGTGA	AAGGTCCAGAGCCAGGAACTC
PKM2	AGGGGCACCCAAGTACATC	TGCCGGAGGAAAGTGAATGAC
PFK1	TGTGGTCCGAGTTGGTATCTT	GCACTTCCAATCACTGTGCC
Nrf2	AGCACATCCAGACAGACACCAGT	TTCAGCGTGGCTGGGGATAT
NQO-1	AGGATGGGAGGTACTCGAATC	AGGCGTCCTTCCTTATATGCTA
HO-1	GAATTCAGCTTGCCACAGGAATTG	TCTACACTAGCTGCATGTTGA

Gene name	Forward primer (5′–3′)	Reverse primer (5′–3′)
β-actin	GGGTCAGAAGGACTCCTATG	GTAACAATGCCATGTTCAAT
Let-7a-5p	TGAGGTAGTAGGTTGTATAGTT	mRQ 3′ Primer
Let-7b-5p	TGAGGTAGTAGGTTGTGTGGTT	mRQ 3′ Primer
Let-7c-5p	TGAGGTAGTAGGTTGTATGGTT	mRQ 3′ Primer
Let-7e-5p	TGAGGTAGGAGGTTGTATAGTT	mRQ 3′ Primer
Let-7f-5p	TGAGGTAGTAGATTGTATAGTT	mRQ 3′ Primer

Bcl-2, B-cell lymphoma 2; Bax, Bcl-2 associated X protein; Caspase-3, cysteinyl aspartate specific proteinase-3; BDNF, brain-derived neurotrophic factor; TRkB, Tyrosine kinase receptor B; CAMK2A, Ca^{2+}/calmodulin-dependent protein kinase II alpha chain; CREB, cAMP response element-binding protein; Synpo, synaptopodin; IRS-1, insulin receptor substrate-1; GULT1, glucose transporter; HK2, hexokinase 2; PKM2, M2 pyruvate kinase; PFK1, phosphofructokinase 1; Nrf2, Nuclear factor erythroid 2-related factor 2; NQO-1, NADPH quinone oxidoreductase-1; HO-1, Heme oxygenase-1; U6 and mRQ 3′ Primer were provided by the kit manufacturer.

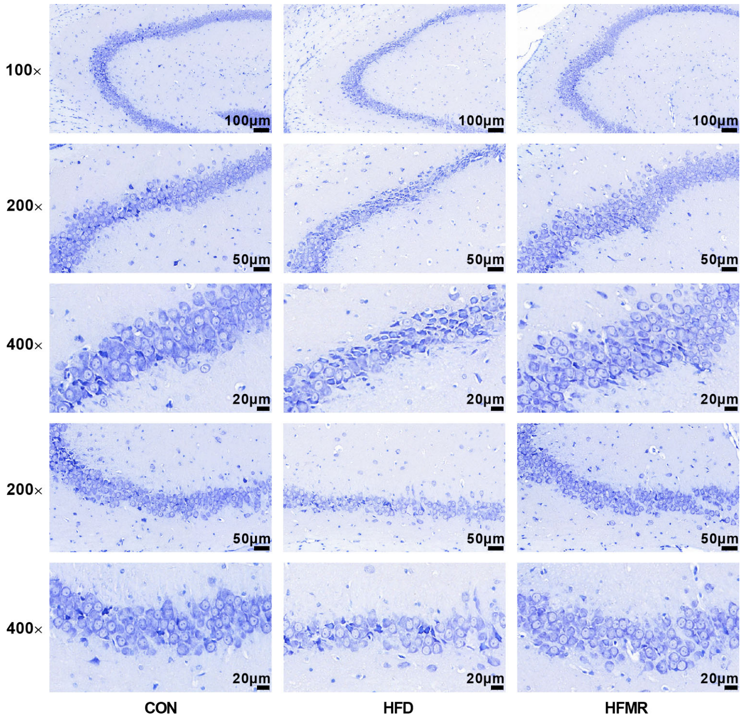

Figure.S1 Effects of MR on neuronal injury in the hippocampus of HFD insulin-resistant mice.

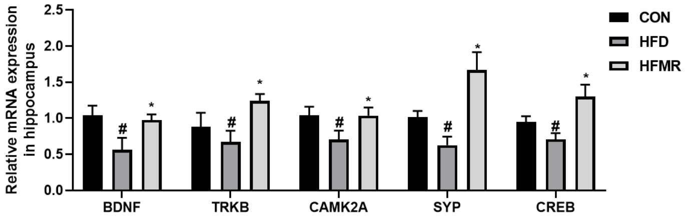

Figure.S2 Effects of MR on learning and memory function related mRNA in the hippocampus of HFD insulin resistance mice. All data are shown as the mean ± SEM (n = 8). # $p < 0.05$, ## $p < 0.01$ (HFD versus CON); * $p < 0.05$, ** $p < 0.01$ (HFMR versus HFD).
